# Comprehensive Analysis of *NAC* Transcription Factors Reveals Their Evolution in Malvales and Functional Characterization of *AsNAC019* and *AsNAC098* in *Aquilaria sinensis*

**DOI:** 10.3390/ijms242417384

**Published:** 2023-12-12

**Authors:** Zhuo Yang, Wenli Mei, Hao Wang, Jun Zeng, Haofu Dai, Xupo Ding

**Affiliations:** 1 Key Laboratory of Research and Development of Natural Product from Li Folk Medicine of Hainan Province, Institute of Tropical Bioscience and Biotechnology, Chinese Academy of Tropical Agricultural Sciences, Haikou 571101, China; yangzhuo1872@163.com (Z.Y.); meiwenli@itbb.org.cn (W.M.); wanghao@itbb.org.cn (H.W.); zengjun@itbb.org.cn (J.Z.); 2 International Joint Research Center of Agarwood, Institute of Tropical Bioscience and Biotechnology, Chinese Academy of Tropical Agricultural Sciences, Haikou 571101, China; 3 Hainan Engineering Research Center of Agarwood, Institute of Tropical Bioscience and Biotechnology, Chinese Academy of Tropical Agricultural Sciences, Haikou 571101, China

**Keywords:** *NAC*, Malvales, evolution, *Aquilaria sinensis*, agarwood, transcriptional regulation, *PKS*

## Abstract

*NAC* is a class of plant-specific transcription factors that are widely involved in the growth, development and (a)biotic stress response of plants. However, their molecular evolution has not been extensively studied in Malvales, especially in *Aquilaria sinensis*, a commercial and horticultural crop that produces an aromatic resin named agarwood. In this study, 1502 members of the *NAC* gene family were identified from the genomes of nine species from Malvales and three model plants. The macroevolutionary analysis revealed that whole genome duplication (WGD) and dispersed duplication (DSD) have shaped the current architectural structure of *NAC* gene families in Malvales plants. Then, 111 *NAC* genes were systemically characterized in *A. sinensis*. The phylogenetic analysis suggests that *NAC* genes in *A. sinensis* can be classified into 16 known clusters and four new subfamilies, with each subfamily presenting similar gene structures and conserved motifs. RNA-seq analysis showed that *AsNACs* presents a broad transcriptional response to the agarwood inducer. The expression patterns of 15 *AsNACs* in *A. sinensis* after injury treatment indicated that *AsNAC019* and *AsNAC098* were positively correlated with the expression patterns of four polyketide synthase (PKS) genes. Additionally, *AsNAC019* and *AsNAC098* were also found to bind with the *AsPKS07* promoter and activate its transcription. This comprehensive analysis provides valuable insights into the molecular evolution of the *NAC* gene family in Malvales plants and highlights the potential mechanisms of *AsNACs* for regulating secondary metabolite biosynthesis in *A. sinensis*, especially for the biosynthesis of 2-(2-phenyl) chromones in agarwood.

## 1. Introduction

Transcription factors (TFs) are the most important cofactors in transcriptional regulation and post-transcriptional modification. These genes bind to specific sequences upstream of the 5′ end of the transcriptional start site of a gene, thereby regulating the expression of the target gene [1]. NAC (NAM, ATAF1/ATAF2 and CUC2) is a gene family of plant-specific TFs that is widely found in plants. NACs were originally characterized in *Arabidopsis thaliana* and petunias, which both contain a highly differentiated transcriptional activation domain at the C-terminal and a conserved DNA-binding domain at the N-terminal [2,3,4,5].

The TF family of *NAC* has been widely studied for its important roles in plant growth and development, especially for apical meristem formation, flower morphogenesis, leaf senescence, the regulation of hormone signaling, cell division, secondary cell wall biosynthesis, lateral root formation, seed germination, fruit ripening and wood formation [6,7,8,9,10]. For example, *GmNAC109* is involved in lateral root formation in soybean [11]. *SlNAC1* controls the biosynthesis of lycopene and ethylene in tomato [12]. *CmNAC-NOR* directly activates the genes that are involved in the biosynthesis of carotenoids, ethylene and abscisic acid, thereby promoting fruit coloration and ripening in melons [13]. In addition, NAC plays an important role in the response to (a)biotic stresses [14,15,16]. For instance, environmental stimuli, such as high temperature, drought, salinity, pathogens and viral infections, can induce NAC expression, which participates in the regulation of plant stress resistance [17,18]. Previous studies have indicated that overexpression of ATAF1, a TF of *NAC*, can enhance resistance against powdery mildew in barley [19] and that *OsNAC17* can enhance drought tolerance in rice [20]. Further, the *LpNAC9* and *LpNAC17* genes in lily (*Lilium pumilum*) were significantly expressed under abiotic stresses, such as drought, low temperatures, and salt stresses [21]. Additionally, *NAC13*, cloned from poplar (*Populus* L.), enhanced drought and salt tolerance in the plants [22]. Similarly, *BpNACs* mediated the low-temperature stress response of *Betula* plants [23].

NACs also contribute to leaf senescence. *NtNAC080* in tobacco is a positive regulator of leaf senescence, as its overexpression leads to early senescence in *Arabidopsis* leaves [24]. *AtNAP*, *NTL4* [25] and *MtNAC96* [26] in *Arabidopsis* also exhibit a similar regulatory effect. Thus, the NAC family plays pivotal roles in various processes of plant growth and development and in response to environmental stresses. In recent years, *NACs* have been elaborately studied in medicinal plants. Twelve *SbNACs* are involved in regulating the synthesis of flavonoids in the flowers of *Scutellaria baicalensis* [27]. *PgNAC41-2* positively regulates the biosynthesis of saponins in *Panax ginseng* [28] and the NAC family acts as a regulatory effector in the synthesis of a variety of metabolites under salt stress in *Isatis indigotica* [29]. However, a systematic study on the *NAC* gene family has not yet been clearly defined in Malvales, especially in *Aquilaria sinensis.*

*Aquilaria* species are aromatic and ornamental plants in south China and Southeast Asia. However, what make this species highly valuable is its production of agarwood, a dark-brown resin formed in the stem of *Aquilaria* species after injury or (a)biotic stresses [30,31]. Since antiquity, agarwood has been widely used as a natural spice, due to its favored smell and in traditional medicine, practices documented in the Holy Bible [32]. Agarwood is also a valuable medicinal herb and is used to treat ailments such as pain, stomach problems and vomiting [33,34]. Modern natural chemistry and pharmacology studies have revealed that 2-(2-phenyl) chromones (PECs) and sesquiterpenes are the main representative components in agarwood, whereas PECs are usually considered to be related to the quality of agarwood [35,36,37], but their biosynthesis mechanisms remain unclear. Recent studies have demonstrated that the plant type III PKS in *Aquilaria sinensis* is involved in the biosynthesis of diarylpentanoid, which is a ring-opening precursor of PECs. In previous research, plant type III PKS, chalcone synthase (CHS), has been found to catalyze the first rate-limiting step in the biosynthesis of flavonoids that are highly similar to those structures of PECs [38]. However, the remaining steps of PEC biosynthesis after the formation of the precursor of the C6–C5–C6 scaffold also remain mysterious, let alone type III PKS transcriptional regulation. The formation of agarwood is usually accompanied by the response of *Aquilaria* trees to (a)biotic stresses, and previous studies have indicated that many structural domain proteins of *NAC* are involved in the (a)biotic stress responses in plants, such as against bacteria, fungi, insects, drought, cold, salinity and mechanical damage [39]. These findings suggest that *NAC* might also be involved in agarwood formation, or in the regulation of PEC biosynthesis. Therefore, the characterization and functional analysis of *NAC* genes in *A. sinensis* are crucial for understanding their response to inducer treatment and injury stress and improving understanding of their roles in PEC biosynthesis and agarwood formation.

In this study, 1502 *NAC* genes were identified from the genomes of nine Malvales and three representative model plants. Our evolutionary analysis provides some evidence for an *NAC* gene family expansion occurring during the divergence of Malvales species. A total of 111 *AsNAC* transcription factors were characterized to explore their phylogenetic relationship, chromosome loci, gene structure, conserved motif, duplication types, intraspecific and interspecific synteny between *A. sinensis* and other species, and the transcriptional expression profiles in *A. sinensis* under inducer treatment and injury stresses. Finally, *AsNAC019* and *AsNAC098* were identified as potential candidates for the activation and regulation of PEC biosynthesis during agarwood formation. These systematic results will improve our understanding of the evolution and function of the *NAC* gene family in *A. sinensis* and *Aquilaria* trees’ response to various environmental stresses.

## 2. Results

### 2.1. Identification of the NAC Gene in A. sinensis and Eleven Other Species

To identify *NAC* gene sequences from *A. sinensis* and eleven other plant species (eight Malvales plants and three representative model species), BLAST and HMM searches were performed to identify their candidate genes. A total of 113 NAC protein sequences from *Arabidopsis* and seed sequences of PF01849 from PFAM, were used as query terms. In addition, the authenticity of the NAC structural domains in the candidate genes was further verified by searching the Pfam, CDD, MEME and eggNOG-mapper databases. Finally, a total of 111 *NAC* genes were identified in the *A. sinensis* genome (Table S1). Similarly, a total of 1278 *NAC* genes were identified from other species, including 86 from *Corchorus capsularis*, 81 from *Corchorus olitorius*, 126 from *Dipterocarpus turbinatus*, 229 from *Durio zibethinus*, 153 from *Gossypium raimondii*, 243 from *Hibiscus cannabinus*, 127 from *Hopea hainanensis* and 105 from *Theobroma cacao*, respectively. Overall, 44 *NAC* genes were identified in the *Amborella trichopoda* genome and 84 *NAC* genes were identified in the *Vitis vinifera* genome. *NAC* genes in the genomes of *H. cannabinus* and *D. zibethinus* were significantly more numerous compared to other Malvales species, whereas *C. olitorius* genome had the fewest *NAC* genes. The numbers presented in Table 1 were not corrected by BUSCO values, and the total number of genes of a gene family might be influenced by the quality of the genome assembly [40]. Therefore, the total numbers of *NAC* genes might need to be updated when better genome assemblies are available in the future.

### 2.2. Comparative Evolutionary Analysis of the NAC Family in Malvales

**Figure 1:**
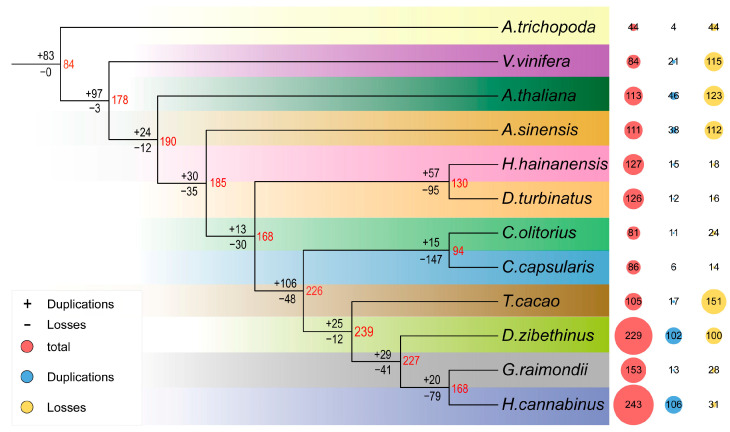
Expansion and contraction of the *NAC* gene families in *A. sinensis* and 11 other species. The + and − represent gene expansion and loss events, respectively; red circles indicate the total number of NAC family members per species; blue and yellow circles indicate the total number of genes gained and lost during evolution, respectively; different color bands indicate cues to different species. The red font in the evolutionary tree indicates the number of ancestor genes.

The expansion and contraction of *NAC* families were examined by reconstructing two phylogenetic trees: a phylogenetic tree of 12 plant species with 302 single-copy genes [41] and an evolutionary tree of all 1502 *NAC* genes. The results showed that a total of 84 ancestral genes were duplicated in the lineages that led to the common ancestor of *A. trichopoda*, *A. thaliana*, *V. vinifera* and Malvales plants. Later, 3 ancestral genes were lost and 97 ancestral genes were further duplicated in the lineages that were common ancestors of Malvales plants, *A. thaliana*, and *V. vinifera* (Figure 1). These events laid the foundation for the *NAC* gene family status in *Arabidopsis*, grape and *A. sinensis*. After two segregations, *A. sinensis* was separated from the other eight Malvales species and the ancestral genes began to expand. The earlier completion of differentiation of *AsNAC* among the Malvales plants suggests that *NAC* genes in *A. sinensis* are relatively conserved in the evolutionary process. Subsequently, the *NAC* ancestral genes of the remaining eight species of Malvales underwent different degrees of expansion before contractions, such as the number of ancestral genes in *H. haianensis*, *D. turbinatus*, *C. capsularis*, *C. olitorius*, *G. raimondii* or *H. cannabinus* (Figure 1).

It is well documented that gene families evolve through whole-genome duplications (WGD), segmental duplications and tandem duplications, which are accompanied by post-duplication diversification [42]. In this study, the duplication types of *NAC* genes in 12 species were examined (Table 1). The proportion of dispersed duplication and WGD-type NAC duplication were higher in *A. sinensis*. Regarding gene duplication events in other Malvales, *C. capsularis*, *C. olitorius*, *T. cacao*, and *A. sinensis* exhibited the same trends, while *NAC* genes in *D. turbinatus*, *D. zibethinus*, *G. raimondii*, *H. cannabinus*, and *H. hainanensis* were mainly retained as WGD events. This points to a different trend of replicative expansion of the *NAC* gene in *A. sinensis* compared to what is observed in other species. In particular, the number of *NAC* genes in *D. zibethinus* (229) and *H. cannabinus* (243) was almost double that in *A. sinensis*. In these two species, the recent WGD driven expansion accounted for 136 (59%) and 161 (66%) *NAC* genes, respectively. Our results suggest that the duplication types of WGD and dispersion may be the main driving forces for the *NAC* gene family expansion in Malvales plants.

### 2.3. Interspecific Synteny Analysis of AsNAC and NAC from Ten Other Species

**Figure 2:**
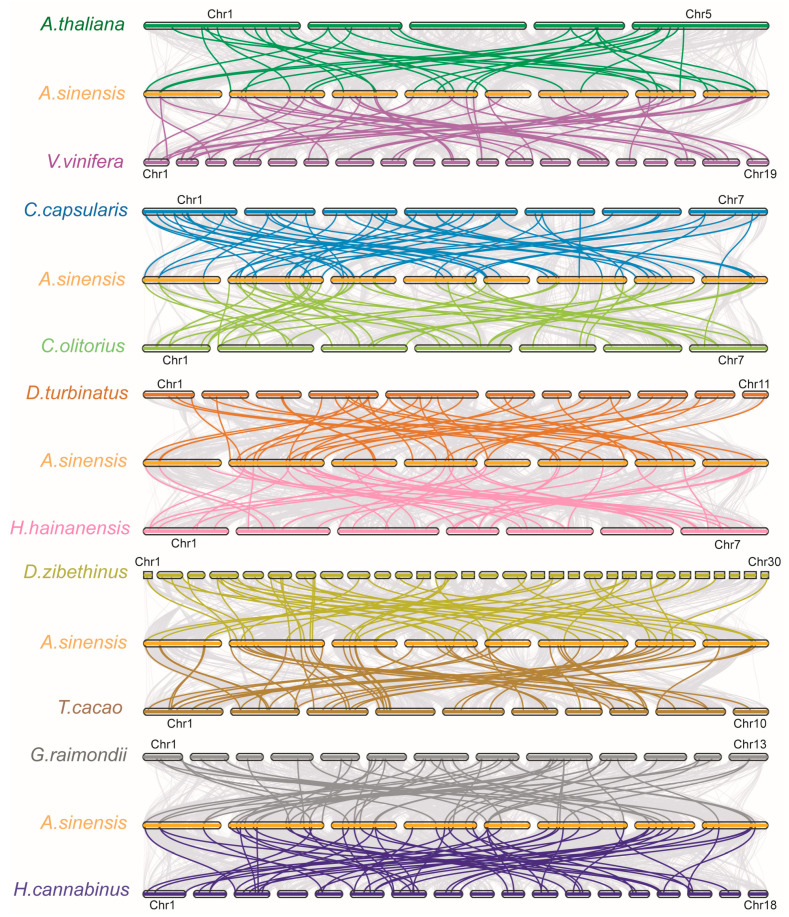
Homology analysis of *NAC* genes between *A. sinensis* and 10 plant species. Color lines indicate *NAC* genes with interspecies synteny with each specie. Gray lines in the background indicate all the collinear genes in the genomes of *A. sinensis* and other plants.

To further explore the potential evolutionary events involving the *NAC* gene family across various species, we constructed the interspecific syntenic maps of *A. sinensis* with the two representative species (*A. thaliana* and grapevine) and eight Malvales plants (Figure 2). A total of 150 *NAC* gene pairs in *D. zibethinus* present the collinearity relationship with those in *A. sinensis*, followed by 130 in *G. raimondii*, 70 in *T. cacao*, 159 in *H. cannabinus*, 63 in *C. capsularis*, 64 in *C. olitorius*, 72 in *V. vinifera*, 76 in *A. thaliana*, 75 in *H. hainanensis* and 78 in *D. turbinatu*. Specifically, 20 *AsNAC* genes presented in a collinear manner in all of the other 10 species, while 37 *AsNAC* genes were not collinear with any of the other 10 species (Figure S1), which suggest that these genes may have been assigned a specific function in *A. sinensis*.

**Figure 3:**
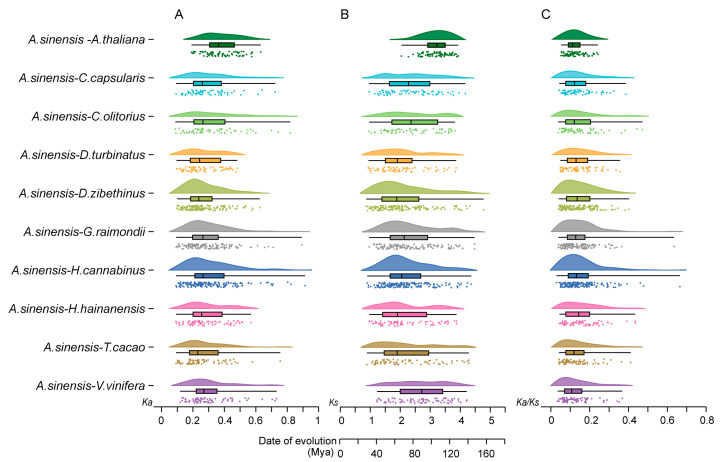
*Ka*, *Ks*, *Ka*/*Ks* and evolutionary time analyses of the *NAC* homologous gene pairs in *A. sinensis* and other species. Different colors represent species. The dot matrix represents the raw data distribution; the peaks are data density curves, which indicated the trend of data concentration. The box-and-line plot represents the data distribution characteristics. (**A**) *Ka*. (**B**) *Ks*. (**C**) *Ka*/*Ks*.

To understand the divergence time and positive selection of orthologous gene pairs, the nonsynonymous substitution rate (*Ka*), synonymous substitution rate *(Ks*), and *Ka*/*Ks* were calculated (Figure 3; Table S3). The *Ka* and *Ks* values of orthologous gene pairs ranged from 0.078 to 0.914 (Figure 3A) and 0.867 to 4.776 (Figure 3B), respectively. All the *Ka*/*Ks* ratios were below 1, indicating the *NAC* gene family from Malvales species is evolving under purifying selection (Figure 3C). Then, the divergence times of orthologous gene pairs between *A. sinensis* and ten other plants were estimated on the basis of the *Ks* values, and we found that the gene pairs might have diverged around 28–159 Mya ago [43,44].

### 2.4. Characterization, Chromosomal Localization and Intraspecific Syntenic Analysis of the AsNAC Genes

**Figure 4:**
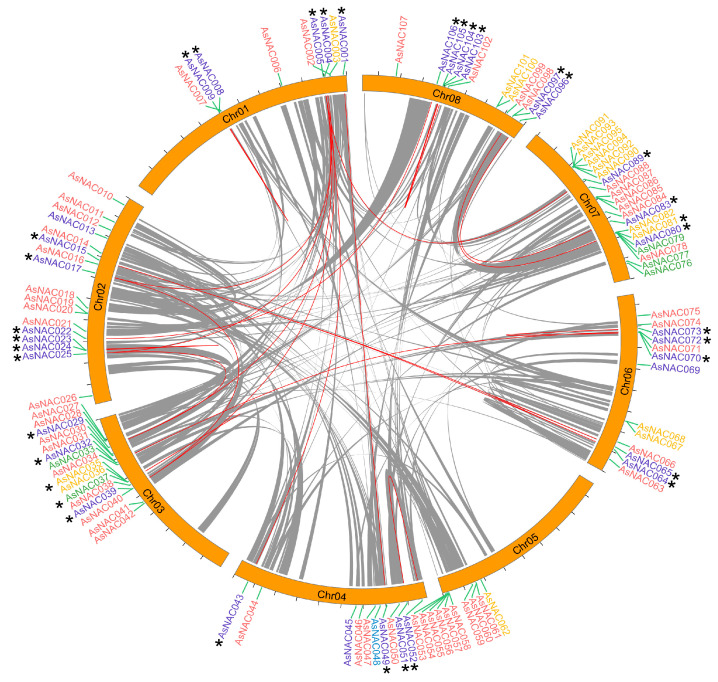
Chromosomal localization and intraspecific homology of the *AsNACs* in *A. sinensis* genome. Gray lines indicate the synteny regions in the *A. sinensis* genome. Red line and * represent the synteny gene pairs of *AsNACs*. Blue, red, green, yellow and purple colors represent the type of duplicates, singleton, duplication dispersed, proximal, tandem and WGD or segmental, respectively.

The physical maps demonstrated that 107 (96%) *AsNAC* genes were localized on 8 chromosomes (Figure 4), and the other 4 *AsNAC* genes were distributed on the unanchored scaffolds, which occupied 3.7% of all *AsNAC* genes. The ORF lengths of *AsNACs* were distributed from 210 bp (*AsNAC087*) to 2286 bp (*AsNAC003*), the amino acid length of AsNAC proteins ranged from 69 (AsNAC087) to 761 (AsNAC003), and the theoretical isoelectric points were between 4.39 (*AsNAC046*) and 9.85 (*AsNAC029*). The molecular weights of AsNAC proteins ranged from 7911.14 Da (*AsNAC087*) to 87,390.79 Da (*AsNAC003*). The average isoelectric point and molecular weight of AsNACs were 6.30 and 39,002.71 Da, respectively. The subcellular localization results showed that most of the *AsNAC* genes were localized in the nucleus (Table S2). In addition, the intraspecific homology of the *AsNAC* gene was analyzed (Figure 4). Twenty-nine intraspecific gene pairs were identified, all of which were derived from WGD, except *AsNAC35* (tandem), *AsNAC37* (proximal) and *AsNAC81* (tandem).

### 2.5. Phylogenetic Analysis of AsNAC and AtNAC

**Figure 5:**
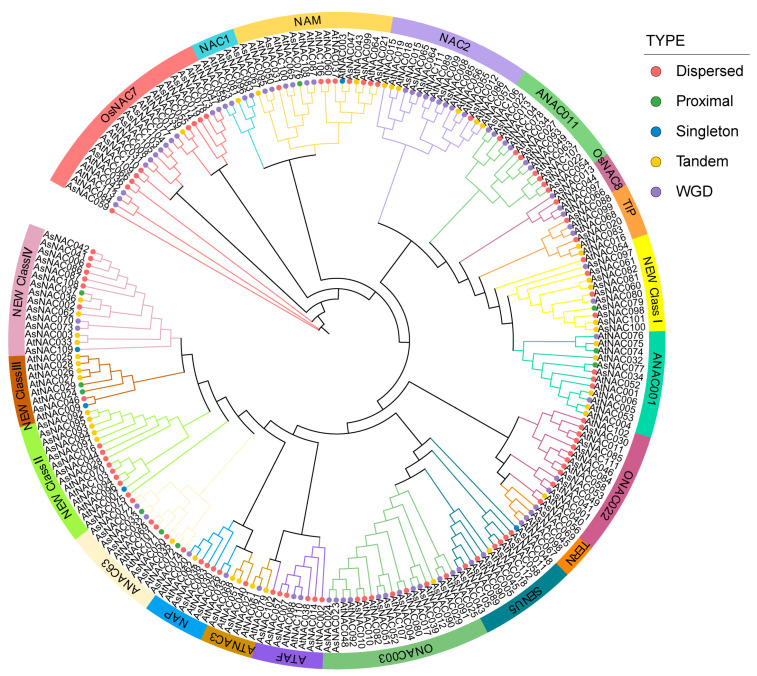
Phylogenetic tree of the relationship of NAC proteins between *A. sinensis* and Arabidopsis. Bands and branches of different colors represent different subfamilies. Solid circles of different colors represent different duplication types.

To investigate their evolutionary relationship and classify the AsNACs, an unrooted evolutionary tree of NAC proteins from *A. thaliana* and *A. sinensis* was generated by IQ-TREE using the best-fit model of VT + F + I + I + R9 with the parameter of MFP (Figure 5). This led to the identification of separate clades of diverse subclasses of *NAC* genes in *A. sinensis*. Then, according to the *NAC* gene family classification system in *Oryza sativa* or *A. thaliana* [2], the independent classification of *NAC* genes in various subgroups of *A. sinensis* was carried out. We found that the *NAC* genes of *A. sinensis* could be categorized into the following 16 types: ANAC001, ANAC011, ANAC63, ATAF, ATNAC3, NAC1, NAC2, NAM, NAP, OsNAC7, OsNAC8, ONAC022, ONAC003, SENU5, TIP and TERN. The ONAC003 subfamily (11) contained the most AsNACs, and the OsNAC8 subfamilies only had one (the lowest) member, *AsNAC013.* Remarkably, 34 *AsNAC* genes were not classified according to the *A. thaliana* subgroups, and consequently formed 4 new subgroups. This indicated a possibility that these AsNACs may have obtained certain specific functions during *A. sinensis* evolution. A total of 53 (48%) *NAC* genes in *A. sinensis* originated from dispersed duplication, 35 (32%) *NAC* genes were duplicated as WGD or segmental, and 2 (2%), 5 (5%) and 16 (14%) genes were derived from singletons, proximal duplication and tandem duplication types, respectively. Among the new subgroups (34 *AsNAC*), only *AsNAC097*, *AsNAC080*, *AsNAC070* and *AsNAC073* were from WGD duplication (12%), indicating that dispersed (44%), proximal (5%), singleton (2%), and tandem (35%) duplication provide greater possibilities for the functional diversity of *AsNAC* genes (Figure 5, Table 1).

### 2.6. Gene Structure and Conserved Motif Analysis of the AsNAC Gene Family

**Figure 6:**
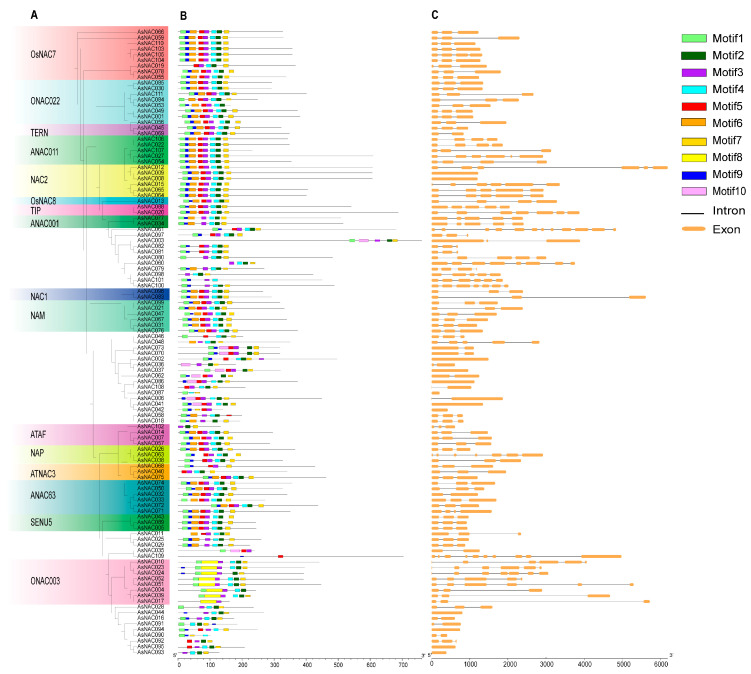
Phylogenetic tree, conserved motifs and gene structure of the AsNACs. (**A**) Phylogenetic relationships were evaluated by IQ-TREE with the best model of VT + F + I + I + R8), where the full-length sequences of the NAC protein of *A. sinensis* were used. (**B**) Ten conserved motifs in AsNAC proteins are represented by the corresponding numbers of colored boxes. (**C**) Exon structures of the AsNAC genes are shown, where yellow boxes indicate exons, and black lines indicate introns.

During the evolutionary course of a species, genetic diversity plays an essential role in shaping phenotype diversity, which enables the plants to adapt to adverse environmental conditions and better use nearby natural resources [6]. We constructed a phylogenetic tree of 111 AsNAC proteins (Figure 6A) and analyzed their conserved motifs, which were visualized by CFVisual software (Figure 6B). The results showed that conserved motifs 1, 2, 3, 4, 7, and 9 were widely distributed in AsNAC proteins. The motifs in the unclassified sequences were diverse and corresponded to the intron–exon boundaries of this subgroup. Specific motifs may be associated with a particular function in different subgroups. All members of the AsNACs, especially in subgroup ONAC003, contained motif 8. Interestingly, motif 10 exists only in the subgroups of the unclassified proteins. Generally, NAC proteins that shared similar motifs were clustered in the same subgroups, indicating functional similarities between these members from the same subgroup.

To investigate the genomic features of *AsNAC*, the gene structures of the *AsNAC* genes were plotted (Figure 6C). The length of the introns of the *AsNACs* ranged from 0 to 5720 bp. No introns were identified in 12 *AsNACs*, most of which (except *AsNAC008* and *AsNAC009*) were in the new subgroups. On the other hand, *AsNAC081* contains the maximum number of 11 introns, followed by *AsNAC014*, which contains nine introns. The large number of introns in these genes could be due to the complex evolutionary history of *AsNACs* in *A. sinensis*. Furthermore, the number of exons in *AsNAC* genes is generally fewer than 10.

### 2.7. Expression Pattern of AsNAC Genes under Agarwood Inducer Treatment

**Figure 7:**
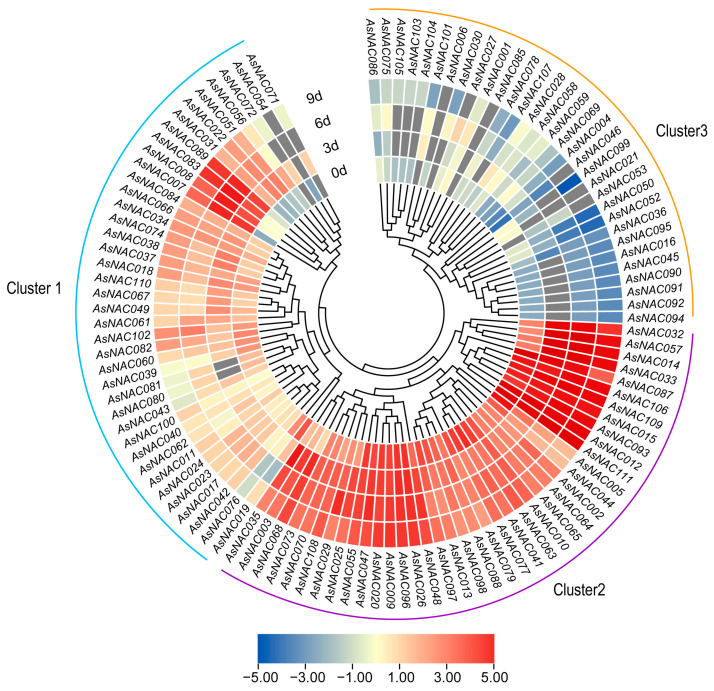
Heat map of RNA-seq-based expression of *AsNAC* genes in response to the treatment with agarwood inducer. The red to purple color represents the log2 scale of the highest to the lowest expression levels. d: days.

To explore the function of *AsNAC* transcription factors in agarwood formation, their expression profiles under inducer treatment were analyzed. A total of 109 *AsNAC* genes exhibited expression patterns and formed three clusters. Cluster 1 contained 35 members with abundant transcription, and these genes showed various expression profiles. Among them, *AsNAC076*, *AsNAC019*, *AsNAC060* and *AsNAC039* were significantly upregulated after the inducer treatment. Cluster 2 contained 45 members, and their expression profiles were significantly higher than the other two clusters. Cluster 3 contained 29 members, and the expression of these genes was significantly downregulated after the inducer treatment (Figure 7).

### 2.8. Expression of AsNAC Genes under Injury Stress

**Figure 8:**
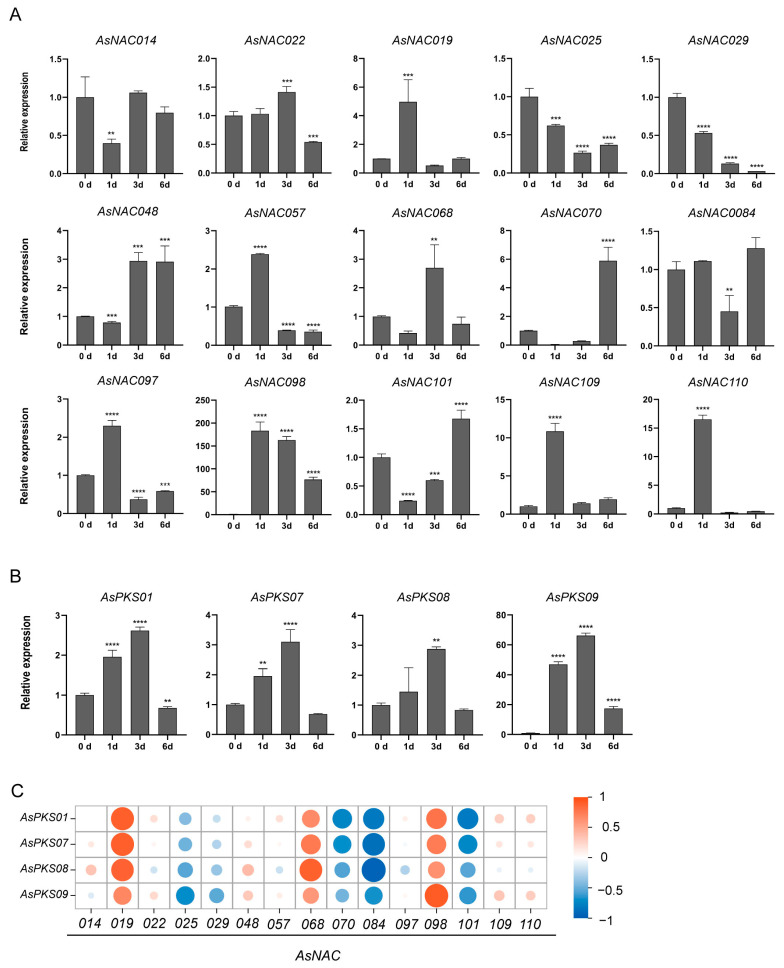
Expression and correlation of *AsNAC* and *AsPKS* genes. (**A**) The relative expression levels of *AsNAC* genes at each time point after treatment were compared with those under normal conditions. (**B**) Relative expression of the *AsPKS* genes. (**C**) Correlation of gene expression of *AsNACs* and *AsPKSs*. Expression at 0 h (no-treatment control) was normalized to 1. Data represent the means ± SD, calculated from three biological replicates. Asterisks (*) indicate significant differences from 0 h after ANOVA; **: *p* < 0.01, ***: *p* < 0.001, ****: *p* < 0.0001.

To explore whether *AsNACs* are involved in regulating PEC biosynthesis, we selected 15 genes that are distributed in each of the branches and evaluated them by using RT-qPCR. These 15 *AsNAC* genes showed variable expression patterns at different stages after injury treatment (Figure 8). This indicated a possibility that these genes may exhibit various functions in a time/stage-dependent manner after injury stress in the stem of *A. sinensis* plants. Eight genes (*AsNAC019, AsNAC022*, *AsNAC057*, *AsNAC097*, *AsNAC098*, *AsNAC097*, *AsNAC109* and *AsNAC110*) were significantly upregulated at 1 day after injury treatment, and then they were downregulated. *AsNAC025* and *AsNAC029* showed a continuous decrease in expression at days 1, 3, 5 and 6 after treatment. *AsNAC025* and *AsNAC029* showed a continuous downward trend in expression at days 1, 3, 5, and 6. *AsNAC048* and *AsNAC101* showed a significant downregulation of expression followed by an upregulation of expression (Figure 8A). Meanwhile, *AsPKS01*, *AsPKS07*, *AsPKS08* and *AsPKS09* were also selected to verify by RT-PCR and their expression patterns were significantly upregulated at days of 1 and 3, and downregulated at day 6 (Figure 8B). A correlation analysis performed between *AsNAC* and *AsPKS* gene expressions suggested that the expressions of *AsNAC019*, *AsNAC068*, and *AsNAC098* showed positive correlations with those of *AsPKS* genes (Figure 8C). These results indicated that *AsNAC019*, *AsNAC068*, and *AsNAC098* might be involved in the regulation of PEC biosynthesis as transcriptional activators to promote the expression of *AsPKS* genes.

### 2.9. Subcellular Localization of AsNAC019, AsNAC068 and AsNAC098

**Figure 9:**
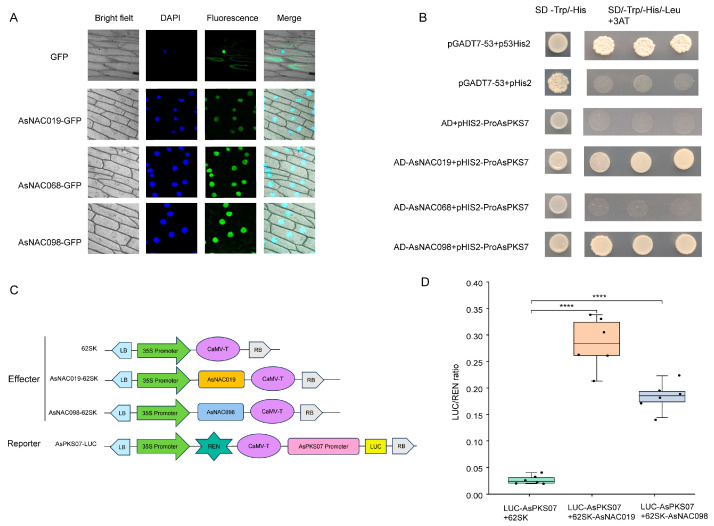
Assays to determine the binding and interaction activities of *AsNAC098* and *AsNAC019* proteins. (**A**) Subcellular localization map of *AsNAC098*, and *AsNAC019* in the onion epidermal cells. (**B**) Yeast one-hybrid experiments of the *AsPKS07* gene promoter with *AsNAC098* and *AsNAC019*. (**C**) Schematic diagrams of the effector and reporter plasmids used in Dual-LUC assays. REN, Renilla luciferase internal reference gene. LUC, firefly luciferase reporter gene. (**D**) Transcriptional activation of *AsNAC019* and *AsNAC098*. A reporter alone, without the effectors, is used as a control. Statistical significance was determined with Student’s *t*-tests: ****: *p* < 0.0001.

To clarify the subcellular localization of *AsNAC019*, *AsNAC068*, and *AsNAC098*, each of them was fused with a GFP tag, transformed into *Agrobacterium tumefaciens* and transfected into onion epidermal cells, respectively. The green fluorescence signals of *AsNAC019*-GFP, *AsNAC068*-GFP and *AsNAC098*-GFP were restricted to the nucleus (Figure 9A), whereas the fluorescent signal carrying the GFP tag alone was distributed throughout the cell. This suggests that *AsNAC019*, *AsNAC068* and *AsNAC098* are nucleus-localized proteins that may provide function in the nucleus (Figure 9A).

### 2.10. Transcriptional Activation of AsPKS07 by Interaction with AsNAC019, AsNAC068 and AsNAC098

To investigate the mechanisms behind *AsNAC*-mediated regulation of *AsPKS07*, *AsNAC019*, *AsNAC068* and *AsNAC098* were further selected to analyze the interactive activation with the promoter of *AsPKS07*, which was abandoned in the functional analysis due to its intron in the previous studies [45], and we identified that it also promoted PEC biosynthesis recently. The assays indicated that *AsNAC019* and *AsNAC098* proteins were able to bind and interact with the *AsPKS07* promoter, whereas *AsNAC068* did not exhibit the same function (Figure 9B).

To verify whether the *AsNAC019* and *AsNAC098* proteins promote the transcription of *AsPKS07*, a dual-luciferase reporter gene assay was performed in *N. benthamiana* leaves. The results indicated that *AsNAC019* and *AsNAC098* significantly increased the initiation activity of the promoter of *AsPKS07* by 9.77- and 5.92-fold, respectively (Figure 9C). These findings suggest that *AsNAC019* and *AsNAC098* might activate the expression of *AsPKS07*.

## 3. Discussion

### 3.1. Identification and Evolution of NAC Transcription Factors

NAC is a unique and slightly larger family of transcription factors in plants. Their numbers are fewer only than those for *bHLH*, *ERF* and *MYB*. For example, 183 *PbNAC* genes were identified in white pear, 180 in apple, 167 *NAC* in cassava; 85 in *Dendrobium nobile*, 90 *NAC* in *Dendrobium catenatum*, 152 in *Glycine max*, 151 in *Oryza sativa*, and 226 such genes were identified in *Triticum aestivum* [46,47,48,49,50]. Recent studies have shown that the genes of the NAC family participate widely in various biological processes [51,52,53,54]. However, most of the studies on the function of *NAC* genes have focused on model plants or crops [55], such as *A. thaliana*, *O. sativa* and *G. max*. However, a systematic study of the NAC family in Malvales has not been conducted, especially in *A. sinensis*. The following numbers of *NAC* transcription factors were identified from nine Malvales plants in this study (Figure 1), including *C. Capsularis* (86), *C. Olitorius* (81), *D. Turbinatus* (126), *D. Zibethinus* (226), *G. raimondii* (153), *H. Cannabinus* (243), *H. haianensis* (127) and *T. cacao* (103). In particular, 111 *NAC* transcription factors were identified from *A. sinensis*, which were named *AsNAC001*–*AsNAC111* (Figure 4). Apparently, the number of members of *AsNAC* gene families is lower than those in species that have undergone the events of WGT and WGD, but more than those in species that have only undergone the WGT event.

Gene duplication is a major evolutionary force for gene family expansion, for example, in the *Arabidopsis* genome, four major duplication events have occurred [56,57]. The number of *AsNAC* and *AtNAC* genes is comparable, and it was hypothesized that similar duplication events might have impacted them during the evolution of *A. sinensis* [58]. Furthermore, the research showed that all the *NAC* transcription factors have evolved from a common ancestor (direct lineage) and underwent many repetitive events during divergent and species-forming (paraphyletic) events that gave rise to different gene functions during plant development and growth [58]. Our analyses suggest that *NAC* is an ancient gene family and that the main drivers of its expansion in Malvales are the recent WGD and dispersed duplication events. Intergenomic covariance analysis showed that a large number of homologous gene pairs existed in *A. sinensis* and other Malvales plants (Figure 2), indicating that the *AsNAC* family was highly conserved during the evolutionary process. The *Ka*/*Ks* values also demonstrated that the purifying selection shaped the landscape of the *NAC* gene family in both the evolution and formation of Malvales plants (Figure 3). Furthermore, the vast majority of homologous genes in the intraspecific homology analysis were derived from the WGD event, which further suggests that the *AsNAC* genes expanded in the recent WGD events. Gene duplications may lead to functional redundancy and similar expression profiles might contribute to the same biological processes, for example, *AsNAC064* and *AsNAC065* share a consistent expression pattern. On the other hand, many homologous genes display differential expression patterns. According to the current theories, this may be due to the fact that duplicated genes have five modes of mutational selection [59].

### 3.2. Potential Function of NAC Genes in A. sinensis

In the phylogenetic tree within multispecies, genes belonging to a single branch tend to have potentially similar function [18]. In our study, the phylogenetic tree constructed by using the NAC proteins of *A. sinensis* and *A. thaliana* showed that homologues of all known *NAC* genes from *A. thaliana* could be found in members of the *AsNAC* gene families (Figure 5). This suggests that the *NAC* gene family existed before the divergence of these two species and therefore the genes in the same subfamily may present similar functions. For instance, the ATAF, NAP, and ATNAC3 subfamilies responded to the stresses [17,60,61], suggesting that *AsNAC* genes in these subfamilies might also contribute to these biological processes. In particular, four new branches containing proteins of unknown function were found in the phylogenetic tree. This may be due to the different environments in which *A. sinensis* and *A. thaliana* evolved, which led to their genetic differentiation. Therefore, the *AsNAC* genes in these new subclades may be related to specific biological traits of *A. sinensis* (Figure 5).

*NAC* transcription factors have highly conserved NAM structural domains at the N-terminus, which are associated with transcriptional regulatory activity and DNA binding [62]. In our analysis, conserved motifs 1 to 6 are annotated as NAM structural domains (Figure 6). Thus, all *AsNAC* genes contain at least one or more of the conserved motifs (1 to 6). In the ONAC003 subfamily, conserved motif 8 was specifically assigned and labeled NAC domain-containing protein suppressor of gamma response 1 (SOG1)-like, which has a conserved role in processes involved in secondary cell wall formation and stress responses. *AtNAC107* and *AtNA082* in the ONAC003 subfamily have been indicated as transcriptional activators that stimulate the expression of the secondary cell wall formation-related transcription factor *MYB46* [63]. Further, the plant-specific transcription factor SOG1 (*AtNAC010*) played a key role in the transmission of ATM and ATR kinase signals that regulate the repair of damaged DNA [64,65]. These results suggest that the *AsNAC* genes in the ONAC003 subfamily may be endowed with similar functions (Figure 6). In addition, a total of 10 *AsNAC* genes (*AsNAC003, 006, 035, 036, 037, 041, 062, 070, 073* and *086*) have a conserved motif 10, and all of them belong to unclassified subfamilies. Combined with covariance analysis, nine *AsNAC* genes, except *AsNAC003*, were not homologous to any *NAC* genes of the other ten species (Figure 6), which implied that these genes could have acquired unique functions during the evolution of *A. sinensis*.

### 3.3. Possible Role of NAC Genes in Secondary Metabolite Synthesis in A. sinensis

The patterns of expression of *AsNAC* genes could provide valuable insights for probing the role of *AsNAC* genes in specific physiological processes. Agarwood is typically produced due to injury in *A. sinensis*. The accumulation of PEC could be increased depending on the treatment of the agarwood inducer. In this study, many *AsNAC* genes showed differential expression under agarwood inducer treatment (Figure 7). Most of the *AsNAC* genes in clusters 1 and 2 exhibited high expression. *AsNAC* genes belonging to ATAF, ATNAC3 and NAP subfamilies appeared in cluster 1 and cluster 3, and exhibited significantly high expression. *AtNAC002*, in the same subfamily, was found to be upregulated under a variety of abiotic stresses and gray mold infection [66]; *AtNAC110* plays a regulatory role in hypoxic stress in plant seedlings [67]. Similarly, *AtNAC088* is thought to be involved in multiple defenses against pathogen infection and in response to injury stimuli [68]. Our results indicate that the *AsNAC* genes in these three subclades may be involved in abiotic stress and defense responses in *A. sinensis*.

The NAC family of transcription factors may also contribute to the regulation of the secondary metabolite biosynthesis of plants. Overexpression of the *PaNAC03* transcription factor in *Picea abies* leads to an independent negative regulation of flavan-3-ol biosynthesis [69]. *SmNAC2* promotes the accumulation of tanshinone I in medicinal ginseng [70], and the *PpNAC* transcription factor known as BLOOD (BL) was identified in peach (*Prunus persica*), which can form a heterodimer with *PpNAC1* and activate the transcription of *PpMYB10.1* to promote the accumulation of anthocyanins in peach fruits [71]. In addition, the *LcNAC002* transcription factor co-activates the expression of *LcSGR* and *LcMYB1* in lychee to help in chlorophyll degradation and anthocyanin biosynthesis [72]. Banana ripening-induced *MaNAC1* is involved in the banana fruit cold response by interacting with *MaCBF1* [73]. The greatest economic value of the *A. sinensis* plant comes from the formation of agarwood, in which PECs are characteristic components whose contents form the benchmark for high-quality agarwood [74]. However, the molecular regulatory mechanisms underlying the formation of PECs are poorly understood.

Biochemically, plant type III PKSs are considered vital and multifunctional enzymes in the secondary metabolite biosynthesis processes of plants in the form of alkaloids, flavonoids or stilbene [75]. Type III PKSs can generate the precursor of PECs by catalyzing malonyl-CoA and 4-hydroxyphenylpropionyl-CoA [45,76]. Our recent studies have demonstrated that the transcription factors in *A. sinensis* were also involved in the regulation of *AsPKS* expression, such as *AsMYB054*, which was the repressor in the transcripts of *AsPKS02* and *AsPKS09* [39]. It is interesting that a syntenic gene pair of *AsbZIP14* and *AsbZIP41* presents a different interaction with *AsPKS* from the same subtype of typical type III PKS, especially for *AsbZIP14*, which activated the expression of *AsPKS08* and repressed the expression of *AsPKS09* [41]. By combining bioinformatics analysis and gene expression data, we also identified two candidate genes that may be associated with the accumulation of PECs (Figure 8). Then, yeast one-hybrid and two-luciferase assays indicated that *AsNAC019* and *AsNAC098* were the activators to regulate the expression pattern of *AsPKS* by binding the specific sequences in the promoter of *AsPKS07* (Figure 9). Taken together, our findings suggest that *AsNACs* may act as transcriptional regulators in *AsPKS* expression to contribute to PEC biosynthesis and agarwood formation, which should be further studied in future research.

## 4. Materials and Methods

### 4.1. Plant Material and Treatment

The samples of *A. sinensis* were collected from Wenchang Nursery Garden (19°54′ N, 110°77′ E). To eliminate physiological and environmental influences, three-year-old trees were selected, and the samples were collected from branches of similar length and age. Holes were drilled into the trunk, approximately 10 cm above the base, 5 mm in diameter and 1 cm deep. Stem segments within 3 cm of the hole were collected after the treatment at days of 0, 1, 3 and 6. Total RNA was immediately isolated with the Plant Total RNA Isolation Kit Plus (FOREGENE, Chengdu, China) according to the manufacturer’s instructions. Then, cDNA was synthesized with the Fastking RT kit (TIANGEN, Beijing, China). The seedlings of *Nicotiana benthamiana* were planted with a photoperiod of 12/12 h (day/night) at 24 °C in the greenhouse at the Institute of Tropical Bioscience and Biotechnology, Chinese Academy of Tropical Agricultural Sciences (19°98′ N, 110°33′). Each treatment contained three biological replicates [41]. The seeds of *N. benthamiana* were obtained from the lab of Dr. Yuan Yao in our institute.

### 4.2. Identification of NAC Gene Family in the Genomes of A. sinensis and 11 Other Plants

The protein sequence of *NAC* genes in *A. thaliana* were collected from the plant transcription factor database (TFDB) [77]. First, the protein sequences of *AtNAC* were used as a query to align the protein sequences of *A. sinensis* by using BlastP [78] with an E-value cutoff of less than 1 × 10^−5^. Meanwhile, the sequences of PF01849, from the PFAM database [79], were also used as seed *NAC* genes to scan the protein database of *A. sinensis* with an HMM search with E-value of 1 × 10^−5^. The results from BLAST and HMM searches were combined to generate the candidate genes of *AsNAC*. These candidates were then verified with CCD [80], MEME [81], InterPro [79], and eggNOG-mapper with default parameters [82].

The genome sequences and files of *A. trichopoda*, *C. capsularis*, *C. olitorius*, *D. turbinatus*, *D. zibethinus*, *G. raimondii*, *H. cannabinus*, *H. hainanensis*, *T. cacao* and *V. vinifera* were collected from NCBI, and the *NAC* transcription factor family in these plants was then identified with the same method as described above for AsNACs. ExPASy software (https://www.expasy.org/) was employed to evaluate the physiochemical properties of *AsNACs*, including the length of ORF and protein, the molecular weight (MW), aliphatic index and instability, and the grand average of hydropathicity (GRAVY) [83].

### 4.3. Expansion and Contraction of NAC Genes

Two evolutionary trees were constructed to illuminate the duplication and loss of the *NAC* gene family in Malvales. Firstly, a phylogenetic tree of species was constructed by using OrthoFinder v 2.5.4 based on the single-copy genes from the 12 species genomes. This tree is the same as that used in our previous study [40,84]. Secondly, a phylogenetic tree of all NAC protein sequences from the 12 species was aligned with MAFFT v.7.310 [85] before constructing an evolutionary tree of proteins using FastTree v 2.1.11 [86]. These two phylogenetic trees were finally analyzed with Notung v 2.9.13 to generate the events of duplications and losses concerning the *NAC* gene family in Malvales species evolution [87].

### 4.4. Identification of Syntenic Genes and Calculation of Ka, Ks and Ka/Ks Values

MCScanX was used to detect gene duplicate types on the basis of BLASTP results from the protein database of each of the species against itself with a cutoff of 1 × 10^−5^ [88]. The identification of potentially homologous gene pairs across multiple genomes was performed with BLASTP with a cutoff of 1 × 10^−5^ [78]. The syntenic gene pairs of *NAC* from *A. sinensis* and ten other species were also identified and visualized by using JCVI v 0.8.4 [89]. The values of *Ka*, *Ks* and *Ka/Ks* were calculated using the MA model in KaKs_calculator 3.0 by using the coding sequences of orthologous gene pairs of *A. sinensis* and the other ten species identified from WGDI v0.6.5 [90]. The divergence periods of each orthologous gene pair were calculated using the formula of T = *Ks*/2*r*, where *Ks* is the synonymous substitution rate per site as mentioned above, and the rate of divergence of the nuclear gene of *r* is set as 1.5 × 10^−8^, which is the synonymous substitutions per site per year of dicotyledonous plants [91].

### 4.5. Phylogenetic Analysis and Chromosomal Localization of the AsNAC Transcription Factors

The MUSCLE program was used to align NAC protein sequences from *A. sinensis* and *A. thaliana* [92,93]. Then, the best model, VT + F + I + I + R9, was selected to construct a phylogenetic tree using IQ-tree v 2.2.0 with the following parameters: -m MFP -B 1000 --bnni -T AUTO [94,95]. The phylogenetic tree was then visualized using iTOL [96]. The gene location of *AsNACs* on the chromosomes of the *A. sinensis* genome and their intraspecific synteny were analyzed with JCVI 0.84 and visualized with Circos v0.9 [97,98].

### 4.6. Gene Structure and Conserved Motif Analyses

The arrangement of exons/introns from *AsNAC* genes was illustrated with GSDS software (http://gsds.gao-lab.org/index.php) [99] using the sequences of genomic DNA and the corresponding CDS region. The 5′ untranslated regions (UTRs) were removed for better comparisons and visualization. The conserved motifs of AsNAC proteins were searched with MEME software (https://meme-suite.org/meme/tools/meme) [81], where the maximum numbers were set as 10 and the optimal motif width was between 30 and 50 amino acids. The conserved motifs were then visualized with the help of CFVisual V2.1.5 software [100].

### 4.7. Real-Time Quantitative PCR (RT-qPCR) Analysis

Fifteen *NAC* genes distributed in each branch were selected on the basis of their differential expression of log2-fold change ≥2 in our previous study for further analysis by RT-qPCR [41]. For RT-qPCR, a histone gene (*His*) was selected as the housekeeping gene [101] and the reaction was run on a CFX96 Real-Time System (Bio-Rad, Hercules, CA, USA). The reaction mixture of a total volume of 20 μL was set as follows: 10 μL 2 × RealUniversal PreMix(TIANGEN), 1 μL of cDNA(100 ng/μL), 0.25 μL primers (0.3 μM) for each of forward and reverse, and 8.5 μL of dd H_2_O. Three biological replicates and two technical replicates were used, and the results were analyzed using the 2^−ΔΔCT^ method [40,102]. The primers used in RT-qPCR are listed in Table S4.

### 4.8. Gene Cloning and Subcellular Localization of AsNAC Genes

The primers for *AsNAC19*, *AsNAC 68*, and *AsNAC 98* were designed and synthesized by Sangon Biotech, Shanghai, China (Table S5). According to the manufacturer’s instructions (MonAmpTM, Monad Biotech, Suzhou, China), the PCR reaction mixture included 25 μL of MonAmpTM 2 × Taq Mix (+Dye), 1 μL forward and reverse primers of each (10 μmol/L), and 1 μL of cDNA (100 ng/μL), and the final volume was 50 μL by dd H_2_O fixed. The reaction parameters were set as follows: pre-denaturation for 2 min at 95 °C, denaturation for 40 s at 95 °C, annealing for 40 s at 55° C, extension for 1 min at 72 °C, 35 cycles, and refolding for 5 min at 72 °C [39].

The sequences of three *AsNAC* plasmids, verified by the method of Sanger sequencing, were used to construct a recombinant vector of pNC-Green-SubN-*AsNAC* [103]. The recombinant vector was transferred into *Agrobacterium tumefaciens* GV3101 receptor cells, which was then infiltrated into the cells of the onion epidermis [39,41]. Transfected onion cells were cultured in the dark at 28 °C for 48 h and the fluorescence values of GFP were attained with a confocal laser scanning microscope (Olympus Corporation, Tokyo, Japan) at 488 nm.

### 4.9. Yeast One-Hybrid (Y1H) Assay

The *Y1H* assay was carried out using the Matchmaker Gold Y1H System (Clontech, Mountain View, CA, USA). The primers used to amplify the promoters of *AsPKS07* (1351 bp) and *AsPKS09* (1272 bp) were described in our previous study [39,40]. These amplified fragments were then integrated into the pHis2.1 vector to form the bait vector, referred to as AsPKSspro-His2.1. Simultaneously, the coding sequence of *AsNACs* was also transformed into the pNC-GADT7 vector via Nimble cloning, generating the prey vector named pNC-GADT7-*AsNAC* [103]. Then, both the prey and bait plasmids were co-transformed into yeast cells of strain Y187. The transformed yeast Y187 strain housed these bait and prey vectors, which were cultivated at 28 °C for 5 days. This cultivation occurred on a selective medium that lacked tryptophan (Trp), histidine (His), and leucine (Leu), while being added to a proper concentration (0.5 mM) of 3-amino-1,2,4-triazole (3AT). Notably, this medium did not contain Trp, Leu, or His (referred to as SD/-TLH + 3-AT).

### 4.10. Dual-Luciferase Assay

The promoter sequences of *AsPKS07* and *AsPKS09* were transformed into the pGreenII 0800-LUC vector to create reporter constructs and the coding sequences of *AsNAC019* and *AsNAC098* were integrated into the pGreenII 62-SK vector as the effector plasmids, also employing the Nimble Cloning system [103]. These dual-luciferase recombinant plasmids were subsequently introduced into the *A. tumefaciens* strain GV3101, which hosted both pGreenII 0800-LUCpAsPKSs and pGreenII 62SK-*AsNAC* mixed in a 1:2 volume ratio. This mixed culture was co-transiented into *N. benthamiana* leaves while the OD600 values reached 0.8 [39]. The luciferase activities were assessed after co-inoculation for 72 h using a dual-luciferase reporter assay kit (Promega, Madison, WI, USA). The ratio of luciferase activities to renilla luciferase activities was then calculated to estimate the transcriptional activity.

## 5. Conclusions

A total of 1502 *NAC* transcription factors were identified in 12 high-quality genomes from the species of Malvales or model plants. Recent WGD might be the driving force behind the current layout of *NAC* in Malvales according to the phylogenetic relationship between species and genes. However, as the basal species in Malvales, *A. sinensis* preserved the medium members of *NAC*, even though this species also experienced the recent WGD event. Therefore, the characterization of 111 *AsNAC* genes was systemically analyzed. The data from the transcriptome and RT-qPCR indicated that *AsNAC* genes may be extensively involved in agarwood formation and stress response. Finally, *AsNAC019* and *AsNAC098* were demonstrated to positively regulate the expression of *AsPKS07* via binding to its promoter. These findings advance our knowledge of the functional and regulatory mechanisms of *NAC* transcription factors in the plant, especially by improving our understanding of their roles in secondary metabolite biosynthesis and environmental stress response.

## Figures and Tables

**Table 1:** Number and duplication types of *NAC* genes in 12 species.

Species	Duplication Type					Total
	Singleton	Dispersed	Proximal	Tandem	WGD	
*A. trichopoda* *	44	/	/	/	/	44
*V. vinifera*	1 (1%)	36 (43%)	6 (7%)	6 (7%)	35(42%)	84
*A. thaliana*	3 (3%)	38 (34%)	5 (4%)	27 (24%)	40 (35%)	113
*A. sinensis*	2 (2%)	53 (48%)	5 (5%)	16 (14%)	35 (32%)	111
*H. hainanensis*	/	18 (14%)	11 (9%)	/	98 (77%)	127
*D. turbinatus*	/	22 (17%)	8 (6%)	3 (2%)	93 (74%)	126
*C. olitorius*	/	47 (58%)	6 (7%)	5 (6%)	23 (28%)	81
*C. capsularis*	1 (1%)	49 (57%)	6 (7%)	5 (6%)	25 (29%)	86
*T. cacao*	2 (2%)	40 (38%)	23 (22%)	12 (11%)	28 (27%)	105
*D. zibethinus*	1 (1%)	18 (8%)	4 (2%)	70 (31%)	136 (59%)	229
*G. raimondii*	1 (1%)	40 (26%)	7 (5%)	10 (7%)	95 (62%)	153
*H. cannabinus*	/	38 (16%)	23 (9%)	21 (9%)	161 (66%)	243
Total	55	399	104	175	769	1502

* The genome of *A. trichopoda* was a scaffold-level genome.

## Data Availability

Data resulting from the database search are available in the Supplementary files.

## Supplementary Materials

The following supporting information can be downloaded at https://www.mdpi.com/article/10.3390/ijms242417384/s1.
