# Supplementary figures and images for: Comprehensive Analysis of NAC Transcription Factors Reveals Their Evolution in Malvales and Functional Characterization of AsNAC019 and AsNAC098 in Aquilaria sinensis
Source: Int J Mol Sci. 2023 Dec 12;24(24):17384. doi: 10.3390/ijms242417384 (PMC10744133; doi:10.3390/ijms242417384)

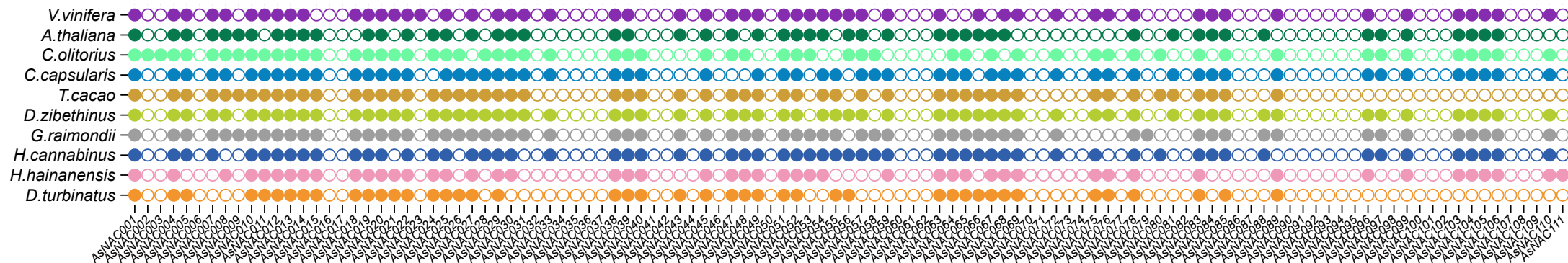

Figure S1. The statistics of interspecies syntenic relationship

Supplement: Supplementary file 1 [file ijms-24-17384-s001.zip › Supplementary Figure S1.pdf]
